# Influenza Vaccination Reduces the Risk of Liver Cancer in Patients with Chronic Kidney Disease: A Nationwide Population-Based Cohort Study

**DOI:** 10.3390/vaccines10122008

**Published:** 2022-11-25

**Authors:** Wen-Rui Hao, Tsung-Yeh Yang, Chun-Chao Chen, Kuan-Jie Lin, Chun-Chih Chiu, Yu-Ann Fang, William Jian, Meng-Huan Lei, Hsien-Tang Yeh, Min-Huei Hsu, Nai-Hsuan Chen, Hung-Chang Jong, Jing-Quan Zheng, Ju-Chi Liu

**Affiliations:** 1 Division of Cardiology, Department of Internal Medicine, Shuang Ho Hospital, Taipei Medical University, New Taipei City 23561, Taiwan; 2 Taipei Heart Institute, Taipei Medical University, Taipei 110301, Taiwan; 3 Division of Cardiology, Department of Internal Medicine, School of Medicine, College of Medicine, Taipei Medical University, Taipei 110301, Taiwan; 4 Graduate Institute of Medical Sciences, College of Medicine, Taipei Medical University, Taipei 110301, Taiwan; 5 Division of Cardiovascular Surgery, Department of Surgery, Shuang Ho Hospital, Taipei Medical University, New Taipei City 23561, Taiwan; 6 Department of Emergency, University Hospitals Cleveland Medical Center, Cleveland, OH 44106, USA; 7 Cardiovascular Center, Lo-Hsu Medical Foundation Luodong Poh-Ai Hospital, Yilan 265010, Taiwan; 8 Department of Surgery, Lotung Poh-Ai Hospital, Luodong 265010, Taiwan; 9 Graduate Institute of Data Science, College of Management, Taipei Medical University, Taipei 110301, Taiwan; 10 Department of Neurosurgery, Shuang Ho Hospital, Taipei Medical University, New Taipei City 23561, Taiwan; 11 Department of General Medicine, Shin Kong Wu Ho-Su Memorial Hospital, Taipei 111045, Taiwan; 12 Graduate Institute of Clinical Medicine, College of Medicine, Taipei Medical University, Taipei 110301, Taiwan; 13 Division of Pulmonary Medicine, Department of Internal Medicine, Shuang Ho Hospital, Taipei Medical University, New Taipei City 23561, Taiwan; 14 Division of Pulmonary Medicine, Department of Internal Medicine, School of Medicine, College of Medicine, Taipei Medical University, Taipei 111045, Taiwan

**Keywords:** chronic kidney disease, influenza vaccination, liver cancer

## Abstract

Previous studies have indicated that influenza vaccination reduces the development of lung cancer. However, the protective effects of influenza vaccination on primary liver cancer in patients with chronic kidney disease (CKD) are unclear. This cohort study identified 12,985 patients aged at least 55 years who had received a diagnosis of CKD between 1 January 2001 and 31 December 2012 from the National Health Insurance Research Database of Taiwan. The patients were classified according to vaccination status. Propensity score matching was used to reduce selection bias. Cox proportional hazards regression analysis was used to evaluate the correlation between influenza vaccination and primary liver cancer in patients with CKD. The prevalence of primary liver cancer was lower in patients with CKD who had received an influenza vaccine (adjusted hazard ratio: 0.45, 95% confidence interval [CI]: 0.35–0.58, *p* < 0.001). The protective effects were observed regardless of sex, age, and comorbidities. Moreover, dose-dependent protective effects were observed. In the subgroup analysis, where the patients were classified by the number of vaccinations received, the adjusted hazard ratios for 1, 2–3, and ≥4 vaccinations were 0.86 (95% CI: 0.63–1.17), 0.45 (95% CI: 0.31–0.63), and 0.21 (95% CI: 0.14–0.33), respectively. In conclusion, influenza vaccination was associated with a lower incidence of liver cancer in patients with CKD.

## 1. Introduction

Chronic kidney disease (CKD) includes many disorders affecting the kidney structure and function. The prevalence and mortality rate of CKD have gradually increased in recent years [1,2]. In Taiwan, the prevalence of CKD is as high as 37.2% among older adults; therefore, it has become an important public health issue [3]. The pathophysiology of CKD is diverse. For example, diabetes may cause diabetic nephropathy due to elevated advanced glycation end products and vasoactive hormones affecting the renin-angiotensin system [4]. In addition, the other risk factors of CKD include high blood pressure, heart disease, and obesity [5]. CKD is associated with many diseases, including cardiovascular diseases and end-stage renal disease, and infection [2]. Furthermore, it has been found that CKD is significantly associated with liver cancer [6,7].

Primary liver cancer is common and is the fourth-ranked cancer-specific cause of death in the world [8,9]. In 2012, the age-standardized incidence rate in Taiwan was much higher than the global average (47.11 and 9.3 cases per 100,000 in Taiwan and worldwide, respectively) [10,11]. Several risk factors have been reported for liver cancer, including hepatitis due to viral infection, aflatoxin exposure, smoking, alcohol consumption, and obesity and diabetes-associated nonalcoholic fatty liver disease [12]. Additionally, the 5-year cumulative incidence rate of primary liver cancer was higher in patients with CKD (0.90) than in the general population (0.85) [6]. Renal dysfunction also negatively affects the prognosis of primary liver cancer [7], which may be due to the shared risk factors of chronic inflammation, compromised immune status, and high uremic concentrations [13].

Seasonal influenza is a highly contagious disease. Globally, approximately 290,000 people die from influenza each year [14,15,16]. The morbidity and mortality associated with influenza are especially high in older adults and individuals with chronic diseases [15]. For instance, the infection of influenza may trigger acute exacerbations and excess hospitalizations in the patients with chronic obstructive pulmonary disease [17]. Furthermore, a previous study found that some virus infections directly and indirectly increase the risk of cancer development, such as the human papillomavirus and hepatitis B, which may induce cervical cancer and hepatocellular carcinoma, respectively [18]. Influenza infection may also have a similar effect. Therefore, the studies investigating the association between influenza and cancer are warranted.

Previous studies have demonstrated that influenza vaccination reduces the risk of infection, hospitalization, and severe disease outcomes [19]. Furthermore, statistics also showed that vaccination reduces inflammation and oxidative stress; thereby, it decreases the risk of lung cancer in patients with different chronic diseases [20]. The pathogenesis of liver cancer is similar to that of lung cancer; however, whether influenza vaccination reduces the risk of liver cancer is unclear [13]. Therefore, this cohort study was conducted to investigate the effects of influenza vaccination on the incidence of liver cancer in patients with CKD in Taiwan using data from the National Health Insurance Research Database (NHIRD).

## 2. Methods

The National Health Insurance (NHI) program in Taiwan was established in 1995; it provides comprehensive healthcare coverage for more than 98% of the population [14]. In this study, we obtained the research data from the NHIRD (2001–2012). The characteristics such as age, sex, or healthcare costs were not statistically significantly different between the sample group and all the enrollees. To ensure patient privacy, all personal information from the NHIRD is delinked and deidentified; furthermore, written agreement declaring that they have no intention of obtaining information that could violate the privacy of patients or care providers must be signed by the researchers using the data. The study was approved by the Joint Institutional Review Board of Taipei Medical University (approval no. N201804043).

### 2.1. Patient Selection Process and the Primary Endpoint

**Figure 1:**
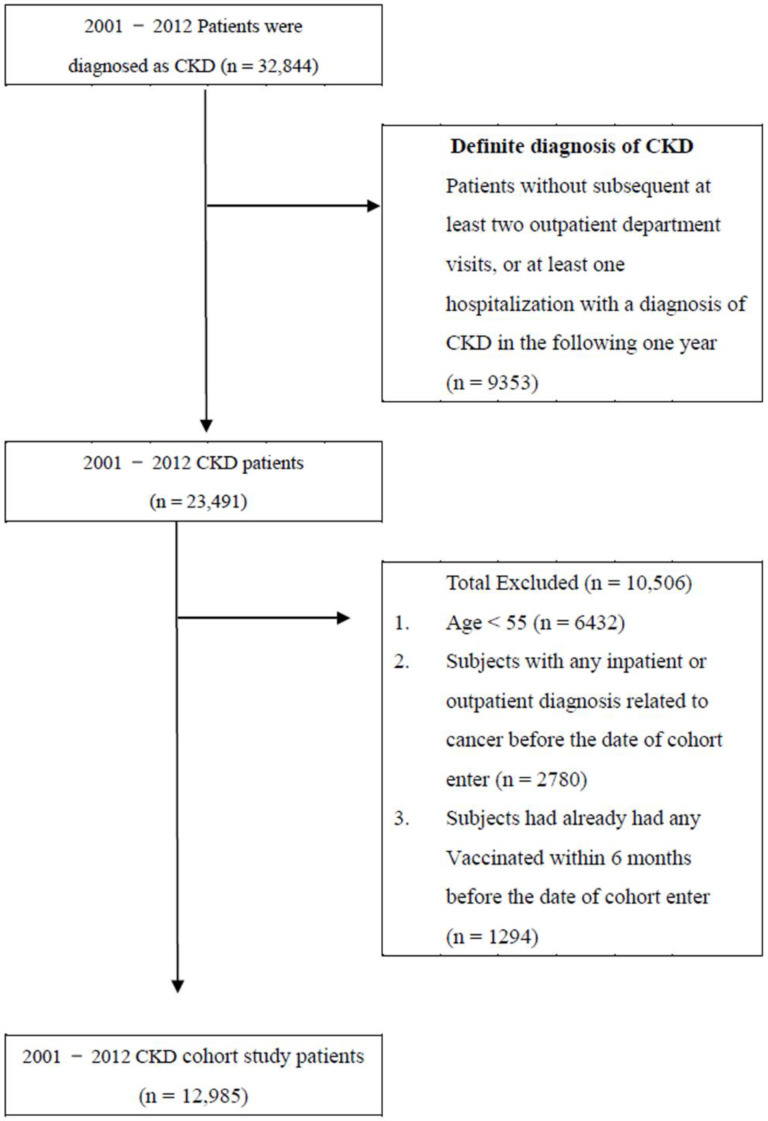
Data selection Process.

The study cohort comprised all patients diagnosed with CKD (according to International Classification of Diseases, Ninth Revision, Clinical Modification [ICD-9-CM] code 585.X) over a 12-year period (*n* = 32,844) from 1 January 2001 to 31 December 2012. We excluded patients without at least two outpatient department visits or at least one hospitalization in which the diagnosis was CKD in the following year (*n* = 9353) due to the uncertainty of their CKD diagnoses. To ensure that no patient had cancer before enrollment, we included a 1-year washout period starting on January 1, 2000. In addition, patients were excluded if they were aged less than 55 years (*n* = 6432) or had received an inpatient or outpatient diagnosis of cancer before the date of enrollment (*n* = 2780) or influenza vaccination within 6 months before the date of enrollment (*n* = 2097). In total, 12,985 patients were included (Figure 1). In Taiwan, the public health policy has been offering influenza vaccinations to high-risk individuals (i.e., those with chronic pulmonary diseases, cardiovascular diseases, chronic liver infection, liver cirrhosis, or type 2 diabetes mellitus) aged more than 50 years at no cost since 1998 and to all individuals aged more than 65 years since 2001 [21]. We analyzed the vaccination status by the ICD-9-CM code V048 or by the vaccine drug codes. The incidence of primary liver cancer (ICD-9-CM code 155.X) in CKD patients was indicated as the primary endpoint of our study in the follow-up years. The follow-up ended on 31 December 2012 or when the patients received a new liver cancer diagnosis, withdrew from NHI, were lost to follow-up, or died.

### 2.2. Potential Confounders

The analysis of the potential confounders, such as sociodemographic characteristics (age, gender, urbanization level, and income), presence of comorbidities (Charlson comorbidity index [CCI] score, diabetes mellitus, hypertension, and dyslipidemia), and medication use (statins, metformin, renin-angiotensin-aldosterone system inhibitor [RAASI], and aspirin) was performed for each patient in this cohort.

### 2.3. Statistical Analysis

To estimate the effects of the vaccination and to reduce the selection bias, a propensity score matching was applied in the comparison between the vaccinated and unvaccinated groups by accounting for the covariates in a logistic regression model [22,23]. For categorical and continuous variables, we used the chi-square test and the *t* test, respectively. Subsequently, we calculated the hazard ratios (HRs) and 95% confidence intervals (CIs) using the Cox proportional hazards regression analysis. Furthermore, we evaluated the dose-response effect of influenza vaccination on the incidence of liver cancer. The patients with CKD were categorized into four groups according to their vaccination status: unvaccinated patients, patients with one vaccine dose, patients with two or three vaccine doses, and patients with four or more vaccine doses. We also stratified patients by age, sex, comorbidities, and medication use. Finally, we used sensitivity analysis to evaluate the differences and similarities between influenza vaccination and the risk of liver cancer in patients with CKD. All statistical analyses were performed using SPSS Statistics (version 22.0, IBM, Armonk, NY, USA) and SAS (version 9.4, SAS Institute, Cary, NC, USA). Statistical significance was indicated at a *p* value of <0.05.

## 3. Results

This cohort study included 12,985 patients. In total, 5495 (42.31%) patients had had an influenza vaccination, and 7490 (57.68%) patients had not had an influenza vaccination. Age, urbanization level, and income significantly differed between the two groups (Table 1). Additionally, the prevalence of comorbidities, including diabetes (*p* < 0.001), hypertension (*p* < 0.001), and dyslipidemia (*p* < 0.001), was higher among the patients without an influenza vaccine than in patients with an influenza vaccine. By contrast, the use of comorbidity-associated medications, such as statins, metformin, RAASI, and aspirin, was longer in patients with an influenza vaccine (Table 1).

Table 2 presents the incidence of liver cancer in patients with CKD with and without an influenza vaccination. After adjustment for potential confounders, the results revealed that the incidence of liver cancer was significantly lower in patients with an influenza vaccine than in those without an influenza vaccine (adjusted HR: 0.45, 95% CI: 0.35–0.58, *p* < 0.001). In our analysis, the protective effects of an influenza vaccination were observed regardless of sex or age and were predominant among patients aged 55–64 years (adjusted HR: 0.28, 95% CI: 0.17–0.46, *p* < 0.001) and among women (adjusted HR: 0.39, 95% CI: 0.26–0.60, *p* < 0.001, Table 2).

In the sensitivity analysis, we conducted covariate adjustments to assess the association of influenza vaccinations with the risk of liver cancer in different models (Table 3). Furthermore, we used the number of vaccinations to stratify the vaccination groups. The protective effect was still observed in the subgroups of different covariates. In addition, the analysis revealed dose-dependent protective effects; the incidence of liver cancer was significantly lower in patients with ≥4 influenza vaccinations (adjusted HR: 0.21, 95% CI: 0.14–0.33, *p* < 0.001). Among the subgroups of patients with ≥4 vaccinations, the extent of the risk reduction in patients aged 55–64 years (adjusted HR: 0.07, 95% CI: 0.02–0.27, *p* < 0.001) was greater than that in patients aged ≥65 years. The female patients who had received ≥4 vaccinations were significantly protected (adjusted HR: 0.11, 95% CI: 0.04–0.27, *p* < 0.001). The patients with comorbidities were also protected, and the protective effects were notable among those with a CCI score of ≥3 (adjusted HR: 0.17, 95% CI: 0.09–0.32, *p* < 0.001). The patients taking medication for a shorter period were more protected than the patients taking medication for a longer period.

## 4. Discussion

### 4.1. Main Findings

In a previous retrospective cohort study, angiotensin-converting enzyme inhibitors were associated with lower incidence rates of liver cancer and cirrhosis in patients with CKD (CKD-weighted subdistribution HR: 0.15, 95% CI: 0.07–0.33, *p* < 0.001) [24]. The present population-based cohort study showed a similar association: (1) liver cancer was less prevalent among the patients with CKD who had received an influenza vaccination (vs. those who had not); (2) the lower prevalence was more pronounced in women than in men; (3) the protective effects of an influenza vaccination were positively correlated with the number of influenza vaccinations a patient had received; (4) the adjusted HR decreased predominantly in the patients with an influenza vaccination who were aged 55–64 years (vs. patients aged ≥65); and (5) the extent of the risk reduction was predominant in patients with a CCI score of ≥3.

### 4.2. Mechanism of Liver Cancer Development in Patients with CKD

CKD and liver cancer exhibit some common risk factors, including environmental toxins, hepatitis due to viral infection, and metabolic diseases [13]. A previous study found that liver cancer is more prevalent in patients with CKD (vs. patients without CKD); different possible mechanisms were proposed [25]. CKD is a general term for a group of diseases that affect the kidney structure and function; thus, multiple risk factors are involved. In patients with CKD, the accumulation of urea and urea-related metabolites (i.e., *p*-Cresyl sulfate) has been observed [26,27,28,29], and the concentrations of inflammatory cytokines (i.e., IL-1, IL-6, and TNF-α) are higher [30,31,32]. These factors may contribute to liver fibrosis and affect the immune system. Patients with a compromised immune system are more susceptible to hepatitis due to viral infection, and hepatitis may eventually lead to liver cancer development [26,27,28,29,30,31]. In addition, the gut microbiota is altered in patients with CKD. Endotoxins and other bacteria that enter the liver through the bloodstream contribute to the development of liver cancer [13,33,34].

### 4.3. Mechanism Underlying the Association between Incidence of Liver Cancer and Influenza Vaccination

The vaccines against human papillomavirus and hepatitis B have been demonstrated to prevent cervical cancer and liver cancer, respectively. Influenza vaccination was also related to a decline in the incidence of lung cancer in a recent study [20]. Infection with influenza leads to the activation of inflammatory cells and an increase in pro-inflammatory cytokines such as IL-1, IL-6, and TNF-α. These may affect the immune regulation of the liver. In addition, the dysregulation of pro- and anti-inflammatory cytokines can cause liver necrosis and may also activate oncogenic signaling cascades [13,35]. Moreover, influenza infection compromises the immune system by, for example, suppressing the natural killer cell, which plays an important immune role in the liver [36,37]. Additionally, influenza infection alters the gut microbiota composition, similarly to CKD [38,39]. Patients with influenza are more susceptible to microbial attacks, which can lead to the release of endotoxins and bacteria into the bloodstream and ultimately lead to liver fibrosis and hepatoma [13]. The number of times a patient is infected with influenza is positively correlated with the formation of reactive oxygen species, which are associated with cellular damage and the activation of proto-oncogenes [20,40]. Furthermore, influenza infection may decrease kidney function. Influenza infection increases the accumulation of uremic toxins and the risks of liver fibrosis and liver cancer [41]. Consequently, by reducing or preventing the risk factors associated with influenza, influenza vaccination reduces the incidence of liver cancer.

### 4.4. Effect of Influenza Vaccination in Patients with Different Characteristics

In this study, the protective effect of influenza vaccination was predominant in patients aged 55–64 years (vs. patients aged ≥65 years). The reason may be that the immune system declines with age, especially with regard to the activity of the thymus function. In addition, the responses of CD4+ and CD8+ T cells are reduced, and the function of antigen-presenting cells is decreased, in turn affecting the effect of the vaccine [42]. Furthermore, the protective effect of influenza vaccination was predominant in patients with a CCI score ≥3 (vs. patients with a lower score), possibly because higher CCI scores are associated with more fragile liver function. Finally, liver cancer was less prevalent in the vaccinated women than in the vaccinated men. Previous studies have found that estrogen is associated with lower concentrations of serum IL-6 [43]. As mentioned earlier, elevated IL-6 concentrations are a risk factor for liver cancer. Estrogen and influenza vaccines may have synergistic effects.

## 5. Limitations

This study has some limitations. First, the diagnosis of CKD was based on ICD-9-CM codes; the accuracy of the diagnoses was unclear. However, the NHI administration randomly verifies medical records, and we only selected patients with at least two outpatient visits or one hospitalization with a diagnosis of CKD in the following year. Second, the higher level of antibody production may be related to a better prevention effect of liver cancer. However, the NHIRD lacks biochemical data such as the estimated glomerular filtration rate (eGFR) and the level of antibody and demographic data such as smoking habits and alcohol consumption. Because the data of these potential confounders were not available, we used propensity score matching to reduce the bias. Third, to reduce the effects of the influenza vaccination policy in Taiwan, this study only included patients aged ≥55 years. Future studies can be designed to incorporate younger patients. Fourth, we were unable to obtain information on the type of the liver cancer. If we could classify the types of liver cancer, such as hepatocellular carcinoma or cholangiocarcinoma, we might be able to measure the effects of vaccination comprehensively. Fifth, the composition of the flu vaccine may vary from year to year. These components might also affect the protective effect of the vaccine in the study. Finally, although the results of this study were significant, this study was not a prospective randomized blinded study. Additional research is required to investigate the precise cause–effect relationship between influenza vaccination and liver cancer in patients with CKD.

## 6. Conclusions

This is the first population-based cohort study to investigate the effect of influenza vaccination on the incidence of liver cancer in patients with CKD. Influenza vaccination was associated with a lower incidence of liver cancer; therefore, regular and planned vaccination may be suitable for patients with CKD. Further research is warranted to explore the underlying mechanisms. In addition, the patients with liver cirrhosis are also closely related to liver cancer. More research is worthwhile to explore the effect of influenza vaccination in this population.

## Figures and Tables

**Table 1:** Characteristics of the sample population.

	Whole Cohort (*n* = 12,985)		Unvaccinated (*n* = 7490)		Vaccinated (*n* = 5495)		*p* ^a^
	*n*	%	*n*	%	*n*	%	
Age, years (Mean ± SD)	70.98 (9.40)		70.09 (10.26)		72.18 (7.90)		<0.001
55–64	3989	30.72	2877	38.41	1112	20.24	<0.001
65–74	4541	34.97	2139	28.56	2402	43.71	
≥75	4455	34.31	2474	33.03	1981	36.05	
Gender							
Female	5712	43.99	3333	44.50	2379	43.29	0.172
Male	7273	56.01	4157	55.50	3116	56.71	
CCI Index ^+^							
0	1491	11.48	876	11.70	615	11.19	0.013
1	2043	15.73	1166	15.57	877	15.96	
2	2876	22.15	1589	21.21	1287	23.42	
≥3	6575	50.64	3859	51.52	2716	49.43	
Diabetes							
No	6310	48.59	3355	44.79	2955	53.78	<0.001
Yes	6675	51.41	4135	55.21	2540	46.22	
Hypertension							
No	2555	19.68	1387	18.52	1168	21.26	<0.001
Yes	10,430	80.32	6103	81.48	4327	78.74	
Dyslipidemia							
No	6337	48.80	3386	45.21	2951	53.70	<0.001
Yes	6648	51.20	4104	54.79	2544	46.30	
Statin							
<28 days	7972	61.39	4786	63.90	3186	57.98	<0.001
28–365 days	2683	20.66	1576	21.04	1107	20.15	
>365 days	2330	17.94	1128	15.06	1202	21.87	
Metformin							
<28 days	10,266	79.06	6045	80.71	4221	76.82	<0.001
28–365 days	1331	10.25	804	10.73	527	9.59	
>365 days	1388	10.69	641	8.56	747	13.59	
RAASI							
<28 days	4114	31.68	2792	37.28	1322	24.06	<0.001
28–365 days	3751	28.89	2344	31.30	1407	25.61	
>365 days	5120	39.43	2354	31.43	2766	50.34	
Aspirin							
<28 days	6715	51.71	4478	59.79	2237	40.71	<0.001
28–365 days	3149	24.25	1702	22.72	1447	26.33	
>365 days	3121	24.04	1310	17.49	1811	32.96	
Level of Urbanization							
Urban	8785	67.65	5350	71.43	3435	62.51	<0.001
Suburban	2806	21.61	1488	19.87	1318	23.99	
Rural	1394	10.74	652	8.70	742	13.50	
Monthly income (TWD)							
0	1596	12.29	901	12.03	695	12.65	<0.001
1–21,000	4486	34.55	2397	32.00	2089	38.02	
21,000–33,300	3788	29.17	1996	26.65	1792	32.61	
≥33,301	3115	23.99	2196	29.32	919	16.72	

^a^ Comparison between unvaccinated and vaccinated. ^+^ CCI index: Charlson comorbidity index; RAASI, renin-angiotensin-aldosterone system inhibitor.

**Table 2:** Risk of liver cancer among unvaccinated and vaccinated in study cohort.

All Group (*n* = 12,985)	Unvaccinated (Total Follow-Up 21,919.2 Person-Years)				Vaccinated (Total Follow-Up 33,990.2 Person-Years)				Adjusted HR † (95% C.I.)
	No. of Patients with Cancer	Incidence Rate (per 10^5^ Person-Years) (95% C.I.)			No. of Patients with Cancer	Incidence Rate (Per 10^5^ Person-Years) (95% C.I.)			
Whole cohort									
All season	162	739.1	(625.3,	852.9)	123	361.9	(297.9,	425.8)	0.45 (0.35, 0.58) ***
Age, 55–64 ^a^									
All season	82	760.1	(595.6,	924.7)	22	244.5	(142.3,	346.7)	0.28 (0.17, 0.46) ***
Age, 65–74 ^b^									
All season	43	701.0	(491.5,	910.5)	63	398.2	(299.9,	496.5)	0.53 (0.35, 0.79) **
Age, ≥75 ^c^									
All season	37	740.4	(501.8,	978.9)	38	414.4	(282.6,	546.1)	0.57 (0.35, 0.91) *
Female ^d^									
All season	60	622.6	(465.1,	780.2)	39	260.3	(178.6,	342.1)	0.39 (0.26, 0.60) ***
Male ^e^									
All season	102	830.4	(669.3,	991.6)	84	441.9	(347.4,	536.4)	0.49 (0.36, 0.67) ***

^a^ Total follow-up 10,787.5 person-year for unvaccinated and 8998.0 for vaccinated. ^b^ Total follow-up 6134.2 person-year for unvaccinated and 15,821.9 for vaccinated. ^c^ Total follow-up 4997.5 person-year for unvaccinated and 9170.3 for vaccinated. ^d^ Total follow-up 9636.6 person-year for unvaccinated and 14,979.9 for vaccinated. ^e^ Total follow-up 12,282.6 person-year for unvaccinated and 19,010.3 for vaccinated. * *p* < 0.05 ** *p* < 0.01 *** *p* < 0.001. C.I.: confidence interval; HR: hazard ratio † Main model is adjusted for age, sex, Charlson comorbidity index, diabetes, hypertension, dyslipidemia, level of urbanization, monthly income in propensity score.

**Table 3:** Sensitivity analysis of adjusted HRs of vaccination in risk reduction of liver cancer in whole season.

	Unvaccinated	Vaccinated			*p* for Trend
		1	2–3	≥4	
	Adjusted HR (95%C.I.)	Adjusted HR (95%C.I.)	Adjusted HR (95%C.I.)	Adjusted HR (95%C.I.)	
Main model †	1.00	0.86 (0.63, 1.17)	0.45 (0.31, 0.63) ***	0.21 (0.14, 0.33) ***	<0.001
Additional covariates ‡					
Main model + Statin	1.00	0.89 (0.65, 1.21)	0.47 (0.33, 0.67) ***	0.23 (0.15, 0.35) ***	<0.001
Main model + Metformin	1.00	0.86 (0.63, 1.18)	0.45 (0.32, 0.64) ***	0.22 (0.14, 0.33) ***	<0.001
Main model + RAA	1.00	0.91 (0.67, 1.23)	0.47 (0.33, 0.67) ***	0.23 (0.15, 0.36) ***	<0.001
Main model + Aspirin	1.00	0.91 (0.67, 1.24)	0.49 (0.34, 0.69) ***	0.24 (0.16, 0.36) ***	<0.001
Subgroup effects					
Age, years					
55–64	1.00	0.50 (0.27, 0.92) *	0.33 (0.16, 0.69) **	0.07 (0.02, 0.27) ***	<0.001
65–74	1.00	1.22 (0.75, 1.99)	0.58 (0.34, 0.97) *	0.23 (0.13, 0.42) ***	<0.001
≥75	1.00	0.98 (0.55, 1.75)	0.42 (0.21, 0.84) *	0.38 (0.18, 0.80) *	0.002
Sex					
Female	1.00	0.90 (0.55, 1.47)	0.36 (0.19, 0.67) ***	0.11 (0.04, 0.27) ***	<0.001
Male	1.00	0.85 (0.57, 1.26)	0.50 (0.33, 0.77) **	0.28 (0.17, 0.45) ***	<0.001
CCI Index ^+^					
0	1.00	1.00 (0.27, 3.65)	0.75 (0.23, 2.44)	0.46 (0.14, 1.54)	0.204
1	1.00	0.57 (0.23, 1.40)	0.65 (0.28, 1.47)	0.23 (0.08, 0.62) **	0.004
2	1.00	0.75 (0.38, 1.47)	0.57 (0.29, 1.11)	0.20 (0.09, 0.45) ***	<0.001
≥3	1.00	0.89 (0.60, 1.32)	0.28 (0.16, 0.49) ***	0.17 (0.09, 0.32) ***	<0.001
Diabetes					
No	1.00	0.88 (0.56, 1.38)	0.54 (0.33, 0.87) *	0.23 (0.13, 0.39) ***	<0.001
Yes	1.00	0.81 (0.53, 1.23)	0.34 (0.20, 0.57) ***	0.20 (0.10, 0.38) ***	<0.001
Dyslipidemia					
No	1.00	0.80 (0.53, 1.19)	0.37 (0.23, 0.59) ***	0.21 (0.13, 0.35) ***	<0.001
Yes	1.00	0.97 (0.60, 1.57)	0.59 (0.35, 1.01)	0.22 (0.10, 0.45) ***	<0.001
Hypertension					
No	1.00	0.80 (0.42, 1.54)	0.52 (0.26, 1.03)	0.20 (0.09, 0.45) ***	<0.001
Yes	1.00	0.87 (0.61, 1.24)	0.42 (0.27, 0.63) ***	0.21 (0.13, 0.35) ***	<0.001
Statin					
<28 days	1.00	0.90 (0.63, 1.28)	0.43 (0.28, 0.65) ***	0.24 (0.15, 0.39) ***	<0.001
28–365 days	1.00	0.80 (0.29, 2.18)	0.77 (0.30, 1.92)	0.24 (0.07, 0.87) *	0.040
>365 days	1.00	0.97 (0.40, 2.36)	0.47 (0.17, 1.32)	0.17 (0.05, 0.60) **	0.003
Metformin					
<28 days	1.00	0.82 (0.57, 1.16)	0.42 (0.28, 0.63) ***	0.19 (0.11, 0.31) ***	<0.001
28–365 days	1.00	1.15 (0.44, 2.99)	0.86 (0.31, 2.38)	0.59 (0.20, 1.80)	0.346
>365 days	1.00	1.05 (0.42, 2.59)	0.42 (0.15, 1.19)	0.24 (0.09, 0.69) **	0.003
RAASI					
<28 days	1.00	0.86 (0.52, 1.41)	0.36 (0.18, 0.70) **	0.09 (0.03, 0.28) ***	<0.001
28–365 days	1.00	1.19 (0.67, 2.11)	0.42 (0.20, 0.88) *	0.36 (0.17, 0.77) **	0.002
>365 days	1.00	0.83 (0.47, 1.44)	0.63 (0.37, 1.07)	0.28 (0.15, 0.50) ***	<0.001
Aspirin					
<28 days	1.00	0.83 (0.54, 1.26)	0.53 (0.33, 0.85) **	0.14 (0.06, 0.31) ***	<0.001
28–365 days	1.00	1.10 (0.59, 2.05)	0.45 (0.21, 0.96) *	0.08 (0.02, 0.36) ***	<0.001
>365 days	1.00	1.15 (0.56, 2.36)	0.54 (0.24, 1.20)	0.57 (0.30, 1.10)	0.043

* *p* < 0.05. ** *p* < 0.01 ***. *p* < 0.001. C.I.: confidence interval; HR: hazard ratio. ^+^ CCI index: Charlson comorbidity index. † Main model is adjusted for age, sex, Charlson comorbidity index, diabetes, hypertension, dyslipidemia, level of urbanization, monthly income in propensity score. ‡ The models were adjusted for covariates in the main model as well as each additional listed covariate.

## Data Availability

The data supporting the findings of this research were sourced from NHIRD in Taiwan. Owing to the legal restrictions imposed by the Government of Taiwan related to the Personal Information Protection Act, the database cannot be made publicly available.
